# Technology Resource, Distribution, and Development Characteristics of Global Influenza Virus Vaccine: A Patent Bibliometric Analysis

**DOI:** 10.1371/journal.pone.0136953

**Published:** 2015-09-15

**Authors:** Ning Chen, Yun Liu, Yijie Cheng, Long Liu, Zhe Yan, Lixin Tao, Xiuhua Guo, Yanxia Luo, Aoshuang Yan

**Affiliations:** 1 School of Public Health, Capital Medical University, Beijing, China, Beijing Municipal Key Laboratory of Clinical Epidemiology, Beijing, China; 2 Beijing Municipal Science and Technology Commission, Beijing, China; 3 School of Management & Economics, Beijing Institute of Technology, Beijing, China; Universidad de Las Palmas de Gran Canaria, SPAIN

## Abstract

Influenza virus vaccine (IVV) is a promising research domain that is closely related to global health matters, which has been acknowledged not only by scientists and technology developers, but also by policy-makers. Meanwhile, patents encompass valuable technological information and reflect the latest technological inventions as well as the innovative capability of a nation. However, little research has examined this up-and-coming research field using patent bibliometric method. Thus, this paper (a) designs the technology classification system and search strategy for the identification of IVV; and (b) presents a longitudinal analysis of the global IVV development based on the European Patent Office (EPO) patents. Bibliometric analysis is used to rank countries, institutions, inventors and technology subfields contributing to IVV technical progress. The results show that the global trends of IVV are a multi-developing feature of variety but an uneven technical resource distribution. Although the synthetic peptide vaccine is a comparatively young field, it already demonstrates the powerful vitality and the enormous development space. With the worldwide competition increasing, all nations especially China should be looking to increase devotion, enhance capability and regard effectiveness of technological innovation.

## Introduction

The influenza virus vaccine (IVV) is a vaccination using a vaccine specifically for a given time to protect against the highly variable influenza virus [1]. Since influenza viruses are continuously changing, vaccination is the best way to protect oneself against the flu. According to the U.S. Centers for Disease Control and Prevention (CDC), seasonal flu vaccines had excellent track records of safety for more than 50 years. For this reason, IVV is commonly considered as a hopeful research domain in the context of pressing global challenges related to health care, fresh air, clean water, and climate change. Many countries and public health organizations have initiated their research and development (R&D) projects in various forms, such as a huge investment in technical platform and infrastructures. The World Health Organization (WHO) established the Global Influenza Surveillance Network in 1952 and launched the Global Action Plan (GAP) for Influenza Vaccines in 2006, aiming at: (1) forming the basis for WHO recommendations on the composition of influenza vaccine; (2) reducing the present global shortage of influenza vaccines for seasonal epidemics and pandemic influenza in all countries of the world. The U.S. CDC and the Food and Drug Administration (FDA) also carefully monitor for any signs that flu vaccines caused. They are working together with the States and local health officials to help prevent the spread of the influenza pandemic throughout the community. Also, the research institutions and enterprises have devoted in the scientific advancement, technology development, and product innovation of IVV.

Patents are the manifestation of the latest and valuable technological inventions, providing a reliable quantification basis for technology or industry development studies [2,3]. As a result, majority of studies have been done using patent bibliometric methods. For example, in an earlier study, 11 technological domains were selected to investigate the science-technology connection in China by means of the scientific non-patent references within patents [4]. Later, another study showed a bibliometric analysis of the patents and scientific publications in the laser cladding field from 1985 to 2007 [5]. In 2010, using supercritical fluids, Yesil-Celiktas and Senyay shed light on the trends of the scientific studies and innovations in the field of particle formation [6]. One year later, patent bibliometric analysis was used for tracking the R&D behavior in the pharmaceutical industry [7]. More recently, researchers had delineated the evolution of major research topics in the area of natural products against cancer with the patent data [8]. Particularly, in the field of nanotechnology, many scholars had characterized the growth, distribution, and trends via an integrated bibliometric analysis using data mining and content visualization tools [3,9–11]. Although the academia has actively promoted IVV research and development, IVV studies using patent bibliometric methods are not yet fully developed.

It is well known that influenza is a serious disease that can lead to hospitalization, sometimes even death. The rapid development, production, and distribution of influenza vaccines could potentially save millions of lives during an influenza pandemic. As a promising research domain with massive potential market, IVV requires extensive research and development to bring out more efficient outcomes. Nevertheless, although various influenza vaccines are being investigated, few articles can be obtained for the global analysis of the evolutionary trends and characteristics of IVV, as well as its subfields.

To be specific, this paper aim to discuss the following two questions.

1. What are the distribution status of the technical resource of IVV and its subfields?
2. What are the technical development characteristics of IVV and its subfields?

The first part of this paper describes the research framework for the longitudinal analysis, including technology classification system, retrieval strategy, database selection and methodology. The second part presents the observations and findings of the evolution and the characteristics of IVV and its subfields from bibliometric analysis. Overall, this study provides an objective reference for both policy makers and scholars to facilitate their decision for the future policy and research of IVV.

## Materials and Methods

### Technology classification system

**Fig 1:**
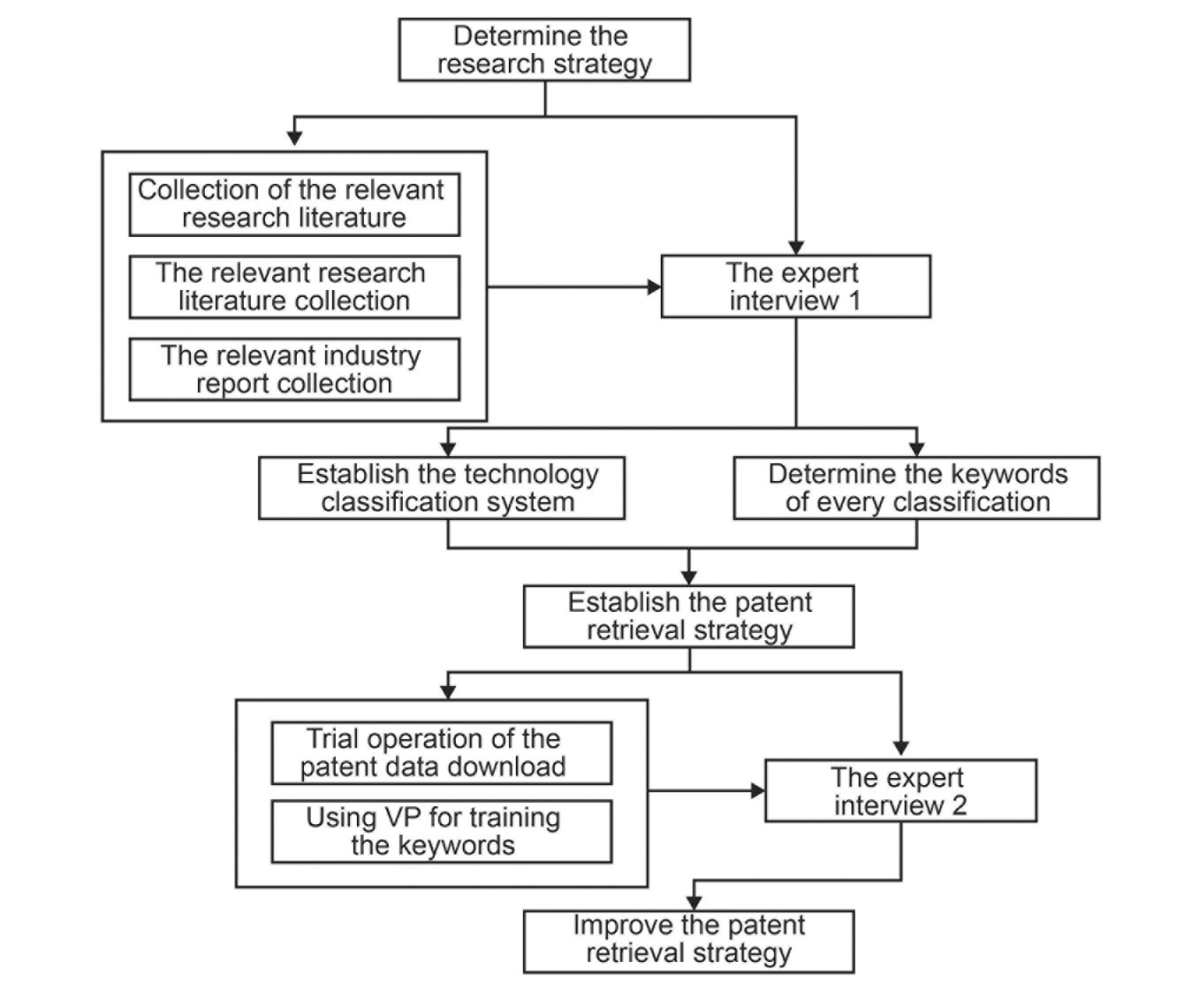
Process for establishing the classification system of IVV.

Since influenza virus was isolated from the body of ferret in 1933, the influenza virus particle surface antigens (hemagglutinin and neuraminidase) mutate continuously, leading to the immunity obtained by human body, hence, IVV technologies constantly update. Therefore, monitoring the IVV technology could clearly sort out the IVV technology classification system, and then grasp the IVV scientific development pedigree. According to the literatures [12,13] and opinions of China’s IVV experts, this paper designed the technology classification system of IVV based on the technical standards of influenza vaccine (Fig 1). IVV can be divided into four major categories, including inactivated vaccine (IV), live attenuated vaccine (LAV), recombinant vaccine (RV), and synthetic peptide vaccine (SPV), whereas it can be further divided into eight subcategories: inactivated virus vaccine, split vaccine, subunit vaccine, live attenuated vaccine, recombinant protein vaccine, recombinant vector vaccine, recombinant DNA vaccine, and synthetic peptide vaccine (Table 1).

**Table 1:** Classification system of IVV technology.

Categories	Subcategories
Inactivated vaccine (IV)	Inactivated virus vaccine
	Split vaccine
	Subunit vaccine
Live attenuated vaccine (LAV)	Live attenuated vaccine
Recombinant vaccine (RV)	Recombinant protein vaccine
	Recombinant vector vaccine
	Recombinant DNA vaccine
Synthetic peptide vaccine (SPV)	Synthetic peptide vaccine

### Database selection

Complex technical information can be organized into logical and understandable statistics by patent data when examining the status of technical resource distribution and development characteristics in a specific technology area. Therefore, the analytical method based on patent data can be used as a useful tool. Founded in 1977, the European Patent Office (EPO) is a federal agency for granting patents to patentee. Both domestic and foreign applicants may submit patent applications to the EPO and thereby request protection of their intellectual property. The possession of patents reflects a country's technological innovation capability. As illustrated in Table 2, the rankings of countries whose patents indexed in the EPO database are roughly proportional to their country’s gross domestic product.

**Table 2:** Countries ranked by the number of EPO-granted patents.

Country	2004–2007	2008–2010	2011–2013	Recent 10 Years	2004–2007
UNITED STATES	1	2	1	1	1
GERMANY	2	1	2	2	2
JAPAN	3	3	3	3	3
FRANCE	4	4	4	4	4
SWITZERLAND	7	5	5	5	7
ITALY	6	6	6	6	6
UNITED KINDOMS↓	5	7	7	7	5
NETHERLANDS	8	8	8	8	8
SWEDEN	9	9	10	9	9
SOUTH KOREA ↑	12	10	9	10	12
CANADA	11	11	11	11	11
FINLAND	10	12	15	12	10
AUSTRIA	13	13	12	13	13
CHINA	14	14	14	14	14
AUSTRALIA	15	15	16	15	15
ISRAEL	16	16	18	16	16
TAIWAN	24	17	13	17	24
DENMARK	17	18	20	18	17

Data compiled by authors for this study.

European countries, which are requested to apply for patents from the EPO first, have a number of the world’s leading vaccine companies such as Novartis, GlaxoSmithKline, Crucell, and so on. Thus, patents related to IVV in the EPO are more representative and comprehensive. Over decades, many laboratories have carried out a series of research based on EPO-granted patents that are considered to have higher technological value than foreign patents and thus can indicate the high quality of invention. For instance, several researchers summarized the characteristics of the patent portfolios in the companies based on survey data from German companies and the EPO [14]. Another study elaborated patenting decisions by firms in relation to the negotiation and signing of the Helsinki and Oslo protocol as part of the Convention on Long-Range Trans boundary Air Pollution using patent data obtained from the EPO [15]. The relationship between patent applications, R&D investment, and capital expenditures from various sources, including the EPO, the EU Industrial R&D Investment Scoreboard, the US Patent and Trademark Office (USPTO), and the World Intellectual Property Organization (WIPO) [16]. In this study, patent data was retrieved from the EPO database.

### Data acquisition and processing

We used a keyword query approach to identifying the IVV-related patent data from the EPO databases. The keywords are based on WHO’s definition of IVV and were previously used in IVV studies [12,13]. Therefore, the retrieved strategy in accordance with the IVV technology classification system was compiled and the patent data for this study was downloaded from the Internet on March 10, 2014. It is worth noting that vaccines can be used in both human and non-human. The VantagePoint software is mainly used for cleaning patents whose title contains “vaccine*”, but whose content has nothing to do with human vaccines, because the downloaded patent data may inevitably include veterinary, poultry or livestock vaccines. After data cleaning up, the collected data included 963 IVV patent records. Since a category may be divided into two or more subcategories, the amount of patent data of subcategory is larger than that of the whole category (Table 3).

**Table 3:** The number of patents of IVV fields and its subfields.

Categories	Subcategories	Number of patents
Inactivated vaccine (IV)	Inactivated virus vaccine	134
	Split vaccine	136
	Subunit vaccine	140
Live attenuated vaccine (LAV)	Live attenuated vaccine	113
Recombinant vaccine (RV)	Recombinant protein vaccine	387
	Recombinant vector vaccine	7
	Recombinant DNA vaccine	178
Synthetic peptide vaccine (SPV)	Synthetic peptide vaccine	19

## Methods

In this paper, a bibliometric analysis of patent data was used during the longitudinal analysis of the global IVV development to identify the evolutionary trends and development characteristics of IVV by subfields of countries, institutions, inventors, and technology and to trace IVV and its technical topic changes over time. Core and high-value patents of IVV were analyzed, as determined by Price's Square Root Law [17] Meanwhile, the citation counts of core patents with high-value patents were compared.

## Results

### Technical resource distribution status of IVV

#### Overall growth trend of patent

**Fig 2:**
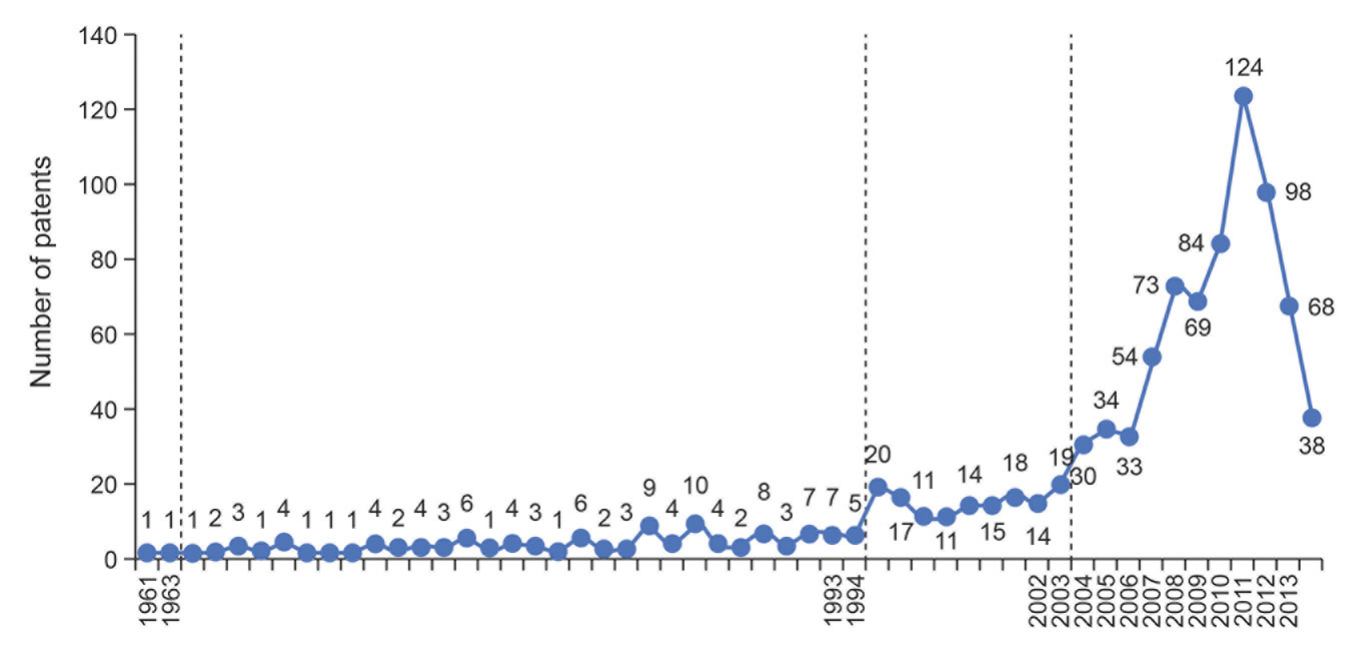
Number of granted patents of IVV by application year.

**Fig 3:**
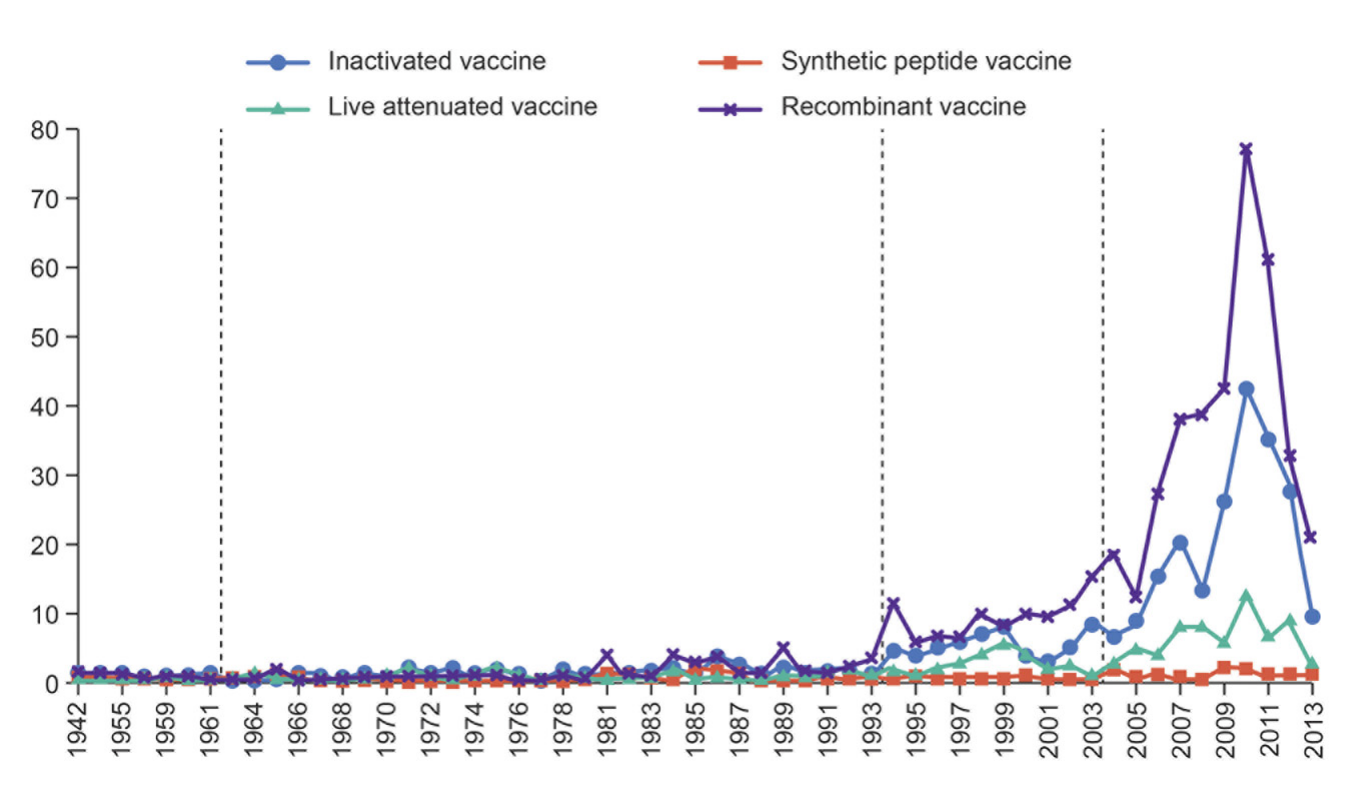
Number of granted patents in four subfields of IVV by application year.

Owing to the emphasis placed on IVV technology R&D expenditure, the world has witnessed spectacular growth in technology innovations of IVV in recent years. The following two figures show the growth of granted patents of IVV and its subfields in terms of their application information (Figs 2 and 3).

According to the global distribution of IVV granted patent by application year, since Geller Ross (from University of Pennsylvania) had obtained the authorization of the first IVV patent in 1942, the amount of IVV patent application shows significantly increasing fluctuation. From 1942 to 1961, the number of IVV patent application was rare, with a total of seven. In the following 30 years (till 1993), the annual patent applications quantity is no more than 10, but with a continuous and stable development. During 1994–2002, the amount increased up to 20, which showed that the global IVV technology had experienced a slow development process. The influenza cases such as H1N1 and SARS in 2003, avian flu in 2005, and H1N1 in 2009 may be the crucial factors that resulted in the increasing number of IVV patent application since 2003. Afterwards, the number of IVV patent application appears to show a downward trend; we speculate the reason is that the patent application per year is close to the monitoring time whereas the patent has not yet been disclosed (all patents downloaded have got authorization; and there is a significant time lag between the date of application and announcement; therefore, the IVV patent data in 2011, 2012, and 2013 is incomplete and hence is just for reference).

As Fig 3 shows, the growth of patent applications in the subfields of IVV has a similar distribution to that of the overall field, which can also be divided into four stages. From 1942 to 1961, other three categories appeared the first patent application except SPV. Obviously, IVV technology was in its early stage. Four categories have similarly showed continued growth until 1993. During 1963–1993, SPV appeared the first patent; RV has annual quantities, which is rich in patent output; the number of IV and LAV patent applications has risen, compared with the previous stage, but they still have no patent applications in some years. From 1994 to 2002, the patent applications of all types of IVV have ushered an increasing trend in addition to SPV. The amount of their patent application is around 5–10 pieces each year. Since 2003, both IV and RV ushered a period of rapid growth of patent applications, reaching a peak of patent applications in 2010. At the meantime, though LAV and SPV also reached a peak in 2010, there are little change compared with the former period, and the number of patents is no more than 10 per year.

In general, the results show that the numbers of patent applications are in the order of RV, IV, LAV, and SPV. Especially, RV’s output in patent achievements is richest. Although the ranking of the four subfields appears to change along with IVV’s slow development, in the fourth stage, namely the last 10 years, the patent applications of four types of vaccines are in full compliance with this order, reflecting the patent achievements and technological innovation strength of each category. Relatively speaking, SPV’s R&D started much later, whose first patent was applied by Israel Yeda Research and Development Co., Ltd. in 1981, and developed more slowly than other three subfields.

#### Origin and target countries

Patent ownership reflects a country’s capability for technological innovation. Similarly, the amount of IVV patents, which come from other countries who applied for and obtained authorization in the EPO, also reflects the level of IVV technology innovation. In this study, the patent’s origin and target countries and organizations are represented by the first two codes (country/area) of each applicant’s country information and patent number (including patent number, family patent number, priority number). Because the World Intellectual Property Organization (WIPO) and the EPO are the important ways for each country to apply for patents, so their statistical analysis is also essential. Table 4 shows the patent application and acceptance conditions of the countries with both IVV patent application and acceptance quantities more than 100.

**Table 4:** Target countries or organizations of IVV patents from main origin countries.

	Target countries or organizations											
Original countries	US	WIPO	CN	EPO	JP	CA	AU	KR	RU	DE	NL	Total
US	226	157	59	116	96	101	106	42	15	27	33	1347
GB	32	28	14	24	25	23	18	8		13	12	390
DE	26	30	9	28	22	21	17	7	1	18	8	313
CN	8	15	242	4	4	2	4	1			1	283
CA	28	23	8	22	16	25	18	9	5	5	9	236
CH	25	22	16	23	16	19	19	11	1	4	11	230
NL	20	17	11	21	10	11	13	4	3	7	8	219
BE	19	11	5	15	13	14	13	2	1	10	3	207
FR	19	18	10	17	11	16	11	6	3	5	6	196
JP	12	15	9	8	52	8	6	4	1	4	1	143
Total	495	435	414	341	298	279	272	158	112	111	105	

Data compiled by authors for this study.

On the whole, all countries participating in the IVV R&D can be divided into three types: (1) IVV patent’s application and acceptance quantities is greater than 100, such as the US, China, Germany, Japan, Canada, etc.; (2) only IVV patent’s application quantity is greater than 100, such as GB, Switzerland, Netherlands, Belgium, France, etc.; (3) only the IVV patent’s acceptance quantity is greater than 100, such as Australia, South Korea, Russia, New Zealand, etc.

The US is a pioneer in IVV technology, and hence it has the largest number of granted patents. As high as 1347, the number of US’s patent applications is nearly three times compared to its acceptance quantity, which indirectly reflects that the US applicants have high level of internationalization and they tend to apply for the protection of a patent with the same technology invention in multiple countries. American applicants who applied for IVV patent by other countries or organizations mainly focus on WIPO, EPO, Australia, Canada, and so on. However, other countries applied for a large number of IVV patents granted by the USPTO, such as the GB, Germany, Canada, Switzerland, and so on. The US has more intense competition in the market for IVV technology, because China, Japan, and Canada belong to the countries whose patent’s application and acceptance quantities are both at the forefront level. Further analysis found that the patents granted by State Intellectual Property Office (SIPO) are mainly from Chinese applicants, and the amount of Chinese IVV patents granted by other countries or organizations is small.

For patent’s application quantity, countries such as GB, Switzerland, Netherlands, Belgium, and France rank the first class. They have a number of the world's leading vaccine companies, for example, Novartis, GlaxoSmithKline, and Crucell; these companies earn the monopoly profit of technology through the patent layout in the global scope. Because a European patent application can specify a multinational protected in each of the EU member countries in accordance with the provisions of the European Patent Convention [18]. These countries apply for patents in EPO in order to save money and simplify the procedures for submission of a separate patent application. The facts mentioned above may be the reasons why patent application in these countries ranked much higher than its acceptance ranking.

For patent’s acceptance quantity, countries such as Australia, South Korea, Russia, and New Zealand rank the first class. It indicates that global IVV applicants give a relatively high degree of attention to these countries, and these countries become major competitive areas of IVV. What is more, if a country’s quantity of patent application is less than its acceptance quantity, it is said that they will have a small number of family patent applications and different patent applications to protect different technological invention. The WIPO is the main destination for each country to apply for international patent applications and hence the granted patents are known as PCT patents. When the applicants are applying for a PCT patent, patent rights related to this PCT patent are valid in each PCT member they specified [19]. At present, there are 435 pieces of PCT patents in IVV, which shows that each country has launched an international competition, which will become more intense in the future.

Note: if a patent is applied by applicants from a number of countries or a patent has obtained authorization by a number of target countries and organizations, the counting will be repeated. For example, a patent’s name is “RECOMBINANT PARAINFLUENZA VIRUS EXPRESSION SYSTEMS AND VACCINES COMPRISING HETEROLOGOUS ANTIGENS DERIVED FROM METAPNEUMOVIRUS”. Its applicants include MEDIMMUNE VACCINES INC from the US and VIRONOVATIVE BV from Netherlands; patent number is US2010297730A1; priority number is EP01200213A, and family patent number is US2003232061A1. Thus, when we analyze this patent, we would make the US-US, US-EP, NL-US, NL-EP for four records plus 1.

#### Technical topics analysis

International Patent Classification (IPC) is a patent classification which is used in patent offices of countries all over the world, and can divided into five levels: department, categories, subcategories, main group, and group. By analyzing the main IPC distribution of IVV patents, the key technical topics can be understood in this technical field, and the key technology and hot technology can be identified. Analysis shows that IVV patent technical topic mainly focus on section A (HUMAN NECESSITIES) and section C (CHEMISTRY, METALLURGY). The number of section A’s patent applications is up to 799 pieces, accounting for 82.88% of all patents, while the number of section C’s patent applications is 490 pieces, accounting for 50.83% of all patents. The remaining are distributed in section G (PHYSICS, 73 pieces, 7.57%) and section B (PERFORMING OPERATIONS, TRANSPORTING, 12 pieces, 1.24%). However, in section H (ELECTRICITY), patents are few (2 pieces). According to the IPC groups corresponding to the number of patents, we found that the technical topics of global IVV patent mainly concentrate on the immune test material orthomyxoviral (A61K39/145), anti-infectives for influenza or rhinovirus (A61P31/16), and virus and its composition, preparation, or purification (C12N7/00) and so on.

**Fig 4:**
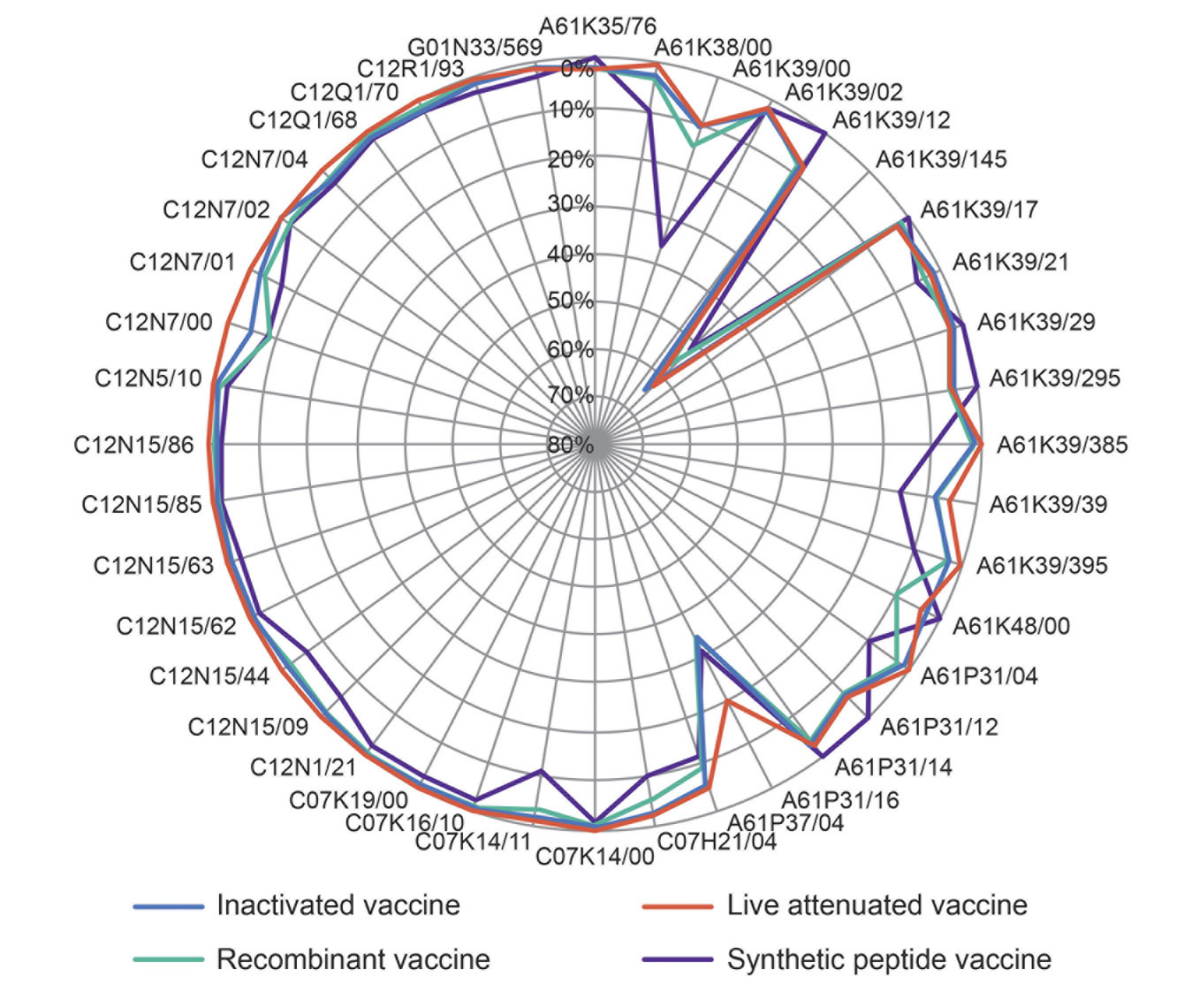
Topic distribution of IVV subfields technology by IPC.

Therefore, the paper produced a radar figure based on the distribution of IPC subgroups of IVV subfields, for the purpose of intuitively displaying the distribution tendency of technical topics in various subfields. To make the comparison more straightforward, we used the proportion of IPC subgroups of subfields as the standardized indicator. Table 5 shows that the IPC distribution of IVV patents is relatively concentrated. In other words, there is an obvious disparity of the patent applications among different IPCs. Some groups accounted for 60% while many others accounted for less than 5%. The radar figure is in reverse order so as to make the distribution tendency of technical topics to be more prominent (Fig 4).

**Table 5:** Major topic distribution of IVV patents.

Rank	IPC	Patent Numbers	Share (%)	Meaning of IPC
1	A61K39/145	571	59.23	Orthomyxoviridae, Viral antigens
2	A61P31/16	322	33.40	Anti-infectives for influenza or rhinoviruses
3	C12N7/00	189	19.61	Viruses; Compositions thereof; Preparation or purification thereof
4	C07K14/11	148	15.35	Orthomyxoviridae, from RNA viruses, Peptides having more than 20 amino acids
5	A61K39/00	137	14.21	Medicinal preparations containing antigens or antibodies
6	C12N7/01	102	10.58	Viruses, modified by introduction of foreign genetic material
7	A61K39/39	99	10.27	Characterised by the immunostimulating additives
8	A61K39/12	90	9.34	Viral antigens
9	A61P37/04	77	7.99	Immunostimulants
10	A61P31/12	73	7.57	Antivirals

As can be seen from the Fig 4, four subfields of IVV have likewise showed a relatively concentrated IPC distribution and an obvious gap in the patent applications among different IPCs. Nevertheless, the IPC groups of four subfields distributed more widely, and 963 pieces patents covered 562 IPC subgroups. Diversity distribution of IVV technical topics showed that, due to the current influenza virus showing the characteristics of diversity and complexity with a development trend [20], demand for the effectiveness and universality of the influenza vaccines are rising as well. Previous finding suggests that inventions in this field are mostly outcomes of interdisciplinary research. Therefore, researchers should expand existing academic areas by making cross fusion between various technologies, in order to generate new perspectives and to get better achievements in vaccine research [21].

The IPC distributions of vaccines in four subfields are roughly similar. There are a large number of patent applications under 5 IPC subgroups, i.e. immune test material orthomyxoviral (A61K39/145), antiinfectives for influenza or rhinoviruses (A61P31/16), virus and its composition, preparation, or purification (C12N7/00), medicinal preparations containing antigens or antibodies (A61K39/00) and characterized by the immunostimulating additives (A61K39/39). In addition, each subfield has respective focus on various IPC subgroups. IV has more patent applications in the subgroup of viral antigens (A61K39/12), antivirals (A61P31/12), recovery or purification of viruses (C12N7/02), etc. The LAV has more patent applications in the subgroup of polyvalent viral antigens (A61K39/295), mixtures of viral and bacterial antigens (C12N7/04), etc. RV has more patent applications in the subgroup of genetically engineered methods to obtain the peptide (C07K14/11), antivirals (A61P31/12), pharmaceutical preparations (A61K48/00), recombinant DNA-technology (C12N15/09), etc. 19 pieces of SPV patent are roughly in two IPC subgroups: medicinal preparations containing antigens or antibodies (A61K) and method of obtaining peptides (C07K).

### Technical development characteristics of IVV

#### Core patents and high-value patents

The bibliometric investigators usually take citation per patent (CPP) and family size (FS) as evaluation indexes. In this report, we defined patents with higher citations as core patents and patents with higher family size as high-value patents.

(1) Citation per patent

Results from the analysis of patent bibliometric show that CPP, times of one patent cited by other patents are the simplest ways to evaluate a patent’s value. CPP cannot only objectively reflect the extent of one patent being used and valued, but can also illustrate the role and status of one patent in the process of academic communication. In short, to some extent, CPP represents the influence level of one patent. Therefore, CPP can used to identify the core patents in IVV.

(2) Family size

Family patents represent the same invention but are patents filed in foreign countries [15]. The role of family patents includes overcoming language barriers, solving the problem of inadequate collection, and learning about the latest technology developments, legal status and economic intelligence of the same invention theme, etc. Family size is as indicative of the value of a specific patent as patent citations [22] and the existence of family members for a given patent as a measure of value has also been recognized by many scholars. Therefore, this paper selected high-value patents in IVV technology innovation based primarily on the FS.

In this study, we used Price’s Square Root Law to determine the core patents. Firstly, N_max_ was defined as the highest number of citation. Through statistical analysis, we found that N_max_ in all IVV patents is equal to 82. Secondly, we defined M as the lowest number of citation in core patents, which means the CPP of core patents were in the range of M to N_max_. According to Price's Square Root Law, M = 6.56, then we determined the patents whose CPP were equal to or higher than 7 as core patents. Therefore, the total number of citation of core patents accounts for about 50% of the total number of citation of all IVV patents. There are 67 pieces of core IVV patents on a global scale. Then, the high-value patents were determined by the same method, and the patents with FS higher than or equal to 7 were selected as high-value patents. The global has a total of 222 pieces of IVV high-value patents.

As showed in Table 6, the patent with highest CPP is applied by the US Health Education & Welfare in 1974, whose CPP is equal to 82 and title is “Temperature-sensitive Recombinant Mutant Viruses and a Process for Producing same”. Among the top 20 core patents, the U.S. has the maximum number of patents, a total of seven, followed by Britain and Japan. Unfortunately, China does not have one patent entering the top 20 CPP. Among the top 20 high-value patents, the U.S. still has the largest number of patents, a total of seven. However, three patents from British held the position of the top 3 CPP and were all applied by GlaxoSmithKline. As can be seen, this company has a more comprehensive patent portfolio across the globe. By comparing the information of the top 20 core patents and high-value patents, we found that there are 39 patents out of 40 patents were distributed in the IPC subclass A61K; further analysis revealed that they were located in IPC main group A61K39, which means “medicinal preparations containing antigens or antibodies”. Thus, the technical innovation in this field has been the most active and valuable part of IVV technology innovation.

**Table 6:** Top 20 core patents and high-value patents for IVV patents.

Rank	Core patents				High-value patents			
	Title	First applicant	Country	Subfields	Title	First applicant	Country	Subfields
1	TEMPERATURE-SENSITIVE RECOMBINANT MUTANT VIRUSES AND A PROCESS FOR PRODUCING SAME	US HEALTH EDUCATION &WELFARE	USA	Recombinant DNA vaccine	PROCESS FOR THE PRODUCTION OF IMMUNOGENIC COMPOSITIONS	GLAXOSMITHKLINE BIOLOG SA	UK	Inactivated vaccine/Live attenuated vaccine/Recombinant vaccine /Synthetic peptide vaccine
2	INFLUENZA VACCINE	DUPHAR INT RES	USA	Inactivated virus vaccine/Recombinant protein vaccine/Recombinant DNA vaccine	VACCINE COMPOSITION	GLAXOSMITHKLINE BIOLOG SA	UK	Live attenuated vaccine
3	VACCINE PREPARATION COMPRISING A BACTERIAL TOXIN ADJUVANT	NAT INST HEALTH	Japan	Subunit vaccine	INTRANASAL INFLUENZA VIRUS VACCINE	GLAXOSMITHKLINE BIOLOG SA	UK	Recombinant DNA vaccine
4	IMMUNOLOGICAL PREPARATIONS	NAT RES DEV	UK	Recombinant protein vaccine	DNA TRANSFECTION SYSTEM FOR THE GENERATION OF INFECTIOUS INFLUENZA VIRUS	STJUDECHILDRENSRES HOSPITAL	USA	Live attenuated vaccine/Recombinant protein vaccine/Recombinant DNA vaccine
5	VACCINE COMPOSITIONS INCLUDING CHITOSAN FOR INTRANASAL ADMINISTRATION AND USE THEREOF	WEST PHARM SERV DRUG RES LTD	UK	Inactivated vaccine	DECREASING POTENTIAL IATROGENIC RISKS ASSOCIATED WITH INFLUENZA VACCINES	CHIRON BEHRING GMBH & CO	USA	Inactivated vaccine
6	INFLUENZA VIRUS VACCINE COMPOSITION	BAXTER AG	Austria	Live attenuated vaccine/Recombinant protein vaccine	IMPFSTOFFE UND DIAGNOSETEST FüR HAEMOPHILUS INFLUENZAE.	SANDOZ AG	Switzerland	Inactivated vaccine/Recombinant protein vaccine/Recombinant DNA vaccine/Synthetic peptide vaccine
7	RECOMBINANT PAPILLOMAVIRUS VACCINE AND METHOD FOR PRODUCTION AND TREATMENT	SMITHKLINE BEECHAM BIOLOG	UK	Recombinant protein vaccine	MANUFACTURE OF INFLUENZA VACCINE	PRAXIS BIOLOG INC	USA	Recombinant DNA vaccine
8	ANIMAL CELLS AND PROCESSES FOR THE REPLICATION OF INFLUENZA VIRUSES	CHIRON BEHRING GMBH & CO	Germany	Recombinant protein vaccine/Recombinant DNA vaccine/Synthetic peptide vaccine	INFLUENZA VACCINE	DUPHAR INT RES	USA	Inactivated virus vaccine/Inactivated vaccine/Recombinant protein vaccine/Recombinant DNA vaccine
9	METHOD AND COMPOSITIONS USEFUL IN PREVENTING EQUINE INFLUENZA	BIOTECH RES PARTNERS LTD	USA	Inactivated vaccine	HIGH MOLECULAR WEIGHT MEDICINE-CONTAINING PREPARATION IN POWDER FORM FOR ADMINISTRATION THROUGH MUCOSA	KIRIN AMGEN INC	Japan	Split vaccine
10	SYNTHETIC HAEMOPHILUS INFLUENZAE CONJUGATE VACCINE	CONNAUGHT LAB	UK	Recombinant protein vaccine/Synthetic peptide vaccine	VACCINE COMPOSITION CONTAINING POLYRIBOSYLRIBITOL PHOSPHATE AND METHOD FOR MAKING SAME	PASTEUR MERIEUX SERUMS VACC	France	Recombinant DNA vaccine
11	INFLUENZA VIRUS SUBUNIT CONJUGATES	CONNAUGHT LAB	USA	Inactivated virusvaccine/Subunit vaccine/Recombinant protein vaccine	VACCINE.	GLAXOSMITHKLINE BIOLOG SA	UK	Recombinant protein vaccine
12	LIPOSOME-CONTAINING INTRANASAL VACCINE FORMULATION	DUPHAR INT RES	Holland	Inactivated vaccine	INFLUENZA HEMAGGLUTININ AND NEURAMINIDASE VARIANTS	MEDLMMUNE LLC	USA	Inactivated vaccine/Live attenuated vaccine/Recombinant vaccine/Synthetic peptide vaccine
13	SYNTHETIC VACCINE AND PROCESS FOR PRODUCING SAME	YEDA RES & DEV	Israel	Synthetic peptide vaccine	No Title Available	PEPTCELL LTD	UK	Split vaccine
14	RECOMBINANT INFLUENZA A VIRUSES	POLYMUN SCIENT IMMUNBIO FORSCH	USA	Recombinant DNA vaccine	PEPTIDE SEQUENCES AND COMPOSITIONS	BAYER AG	Germany	Recombinant protein vaccine
15	LIPOSOMAL INFLUENZA VACCINE COMPOSITION AND METHOD	YISSUM RES DEV CO	USA	Subunit vaccine	IMPFSTOFFHERSTELLUNG VON IMMORTALISIERTEN SUGETIERZELLINIEN	GOETZ REINER	Holland	Inactivated virus vaccine/Inactivated vaccine
16	INFLUENZA VIRUS REPLICATED IN MAMMALIAN CELL CULTURE AND VACCINE PRODUCTION	ST JUDE CHILDRENS RES HOSPITAL	USA	Inactivated vaccine	HBV CORE ANTIGEN PARTICLES WITH MULTIPLE IMMUNOGENIC COMPONENTS ATTACHED VIA PEPTIDE LIGANDS	BIOGEN	USA	Recombinant protein vaccine
17	INFLUENZA VACCINE	NAT INST HEALTH	Japan	Inactivated vaccine	PROCESSES FOR THE REPLICATION OF INFLUENZA VIRUSES IN CELL CULTURE AND THE INFLUENZA VIRUSES OBTAINABLE BY THE PROCESS	NOVARTIS VACCINES	Germany	Inactivated vaccine
18	METHODS AND COMPOSITIONS USEFUL IN PREVENTING EQUINE INFLUENZA	BIOTECH RES PARTNERS LTD	USA	Recombinant protein vaccine/Recombinant DNA vaccine/Synthetic peptide vaccine	LIVE RECOMBINED VACCINES INJECTED WITH ADJUVANT	MERIAL LTD	France	Inactivated vaccine/Live attenuated vaccine/Recombinant vaccine/Synthetic peptide vaccine
19	POLYNUCLEOTIDE FORMULATION AGAINST PATHOLOGIES OF THE HORSE	MERIAL LTD	France	Recombinant protein vaccine	NOVEL METHODS FOR THERAPEUTIC VACCINATION.[ES	PHARMEXA A S	Denmark	Live attenuated vaccine/Recombinant protein vaccine/Recombinant vector vaccine
20	IMMUNOSTIMULANT EMULSION	AVENTIS PASTEUR	France	Recombinant protein vaccine	P DIATRISCHE KOMBINATIONSVAKZINE MIT VERBESSERTER IMMUNOGENIZIT?T JEDER VAKZINE KOMPONENTE.[EN	AMERICAN CYANAMID CO	USA	Recombinant protein vaccine

Data compiled by authors for this study.

**Fig 5:**
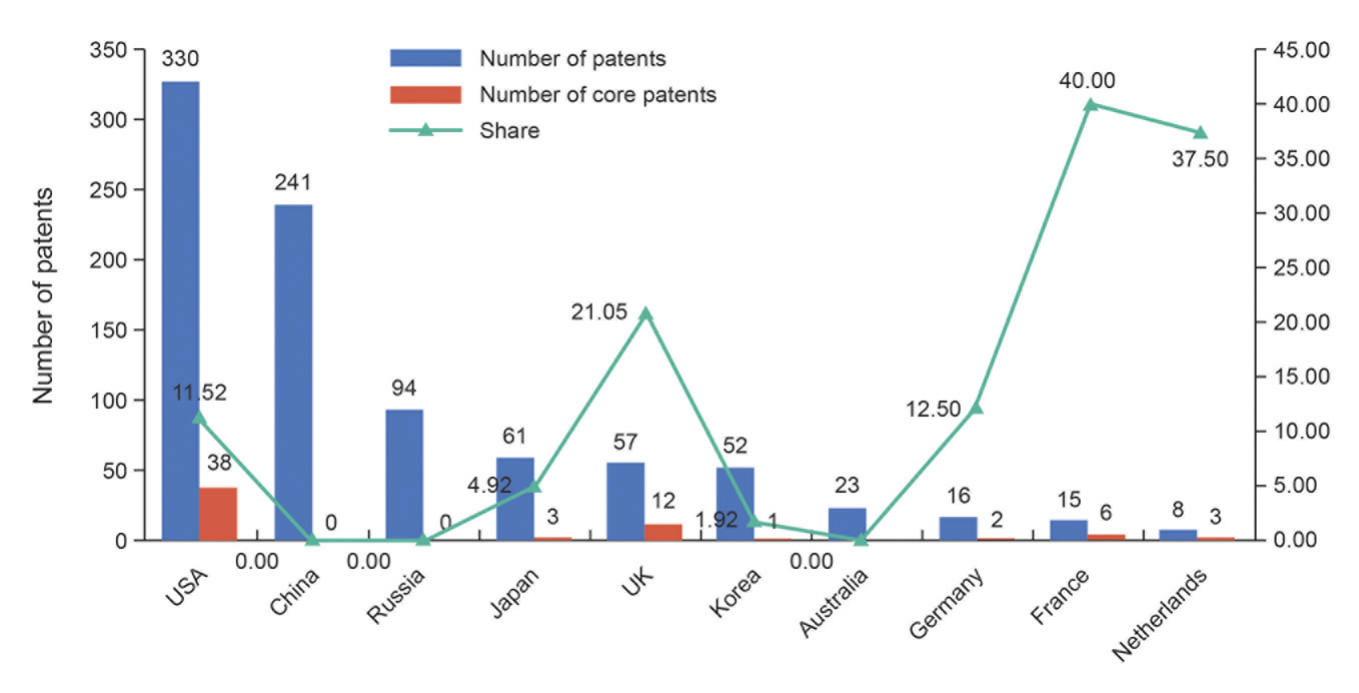
The number of patents and core patents of typical countries.

In this paper, the characteristics of all patents (core patents and high-value patents) of IVV were compared, as showed in Table 7. As can be seen, the core and high-value patents contain more IPC subgroup than the common patents. Hence the value and consequence of a patent are associated with the number of IPC subgroup of that patent; in other words, the technology integration can influence the value and consequence of IVV patents in the certain degree, which confirms the development trend and significance of the technical diversity of above topics. What is more, there is no significant difference between core patents, high-value patents and all patents on the number of applicants and inventors. However, still, it can be clearly found that effective international exchanges and cooperation can promote high-value and high-impact patents generated through relevant statistical data on the number of countries of applicants and inventors. Last but not least, it can be seen that there is certain positive correlation between the high-value and core patents through the data on CPP and FS. High-value patents have higher influence level while core patents have a higher valuation (Fig 5).

**Table 7:** Summary of data statistics for all patents, core patents, and high-value patents of IVV.

	All patents			Core patents			High-value patents		
	Max	Min	Mean	Max	Min	Mean	Max	Min	Mean
Number of IPC subgroup (NIPC)	59	1	4.57	35	1	6.9	59	1	7.56
Number of applicants (NA)	17	1	1.98	8	1	1.65	10	1	2.1
Number of countries of applicants (NCA)	4	1	1.08	4	1	1.15	4	1	1.21
Number of inventors (NI)	23	1	3.93	11	1	3.33	11	1	3.73
Number of countries of inventors (NCI)	6	1	1.14	3	1	1.17	4	1	1.32
Citation per patent(CPP)	82	0	1.56	82	7	20.18	64	0	4.7
Family size (FS)	86	0	5.02	55	0	15.99	86	7	18.07

Data compiled by authors for this study.

**Fig 6:**
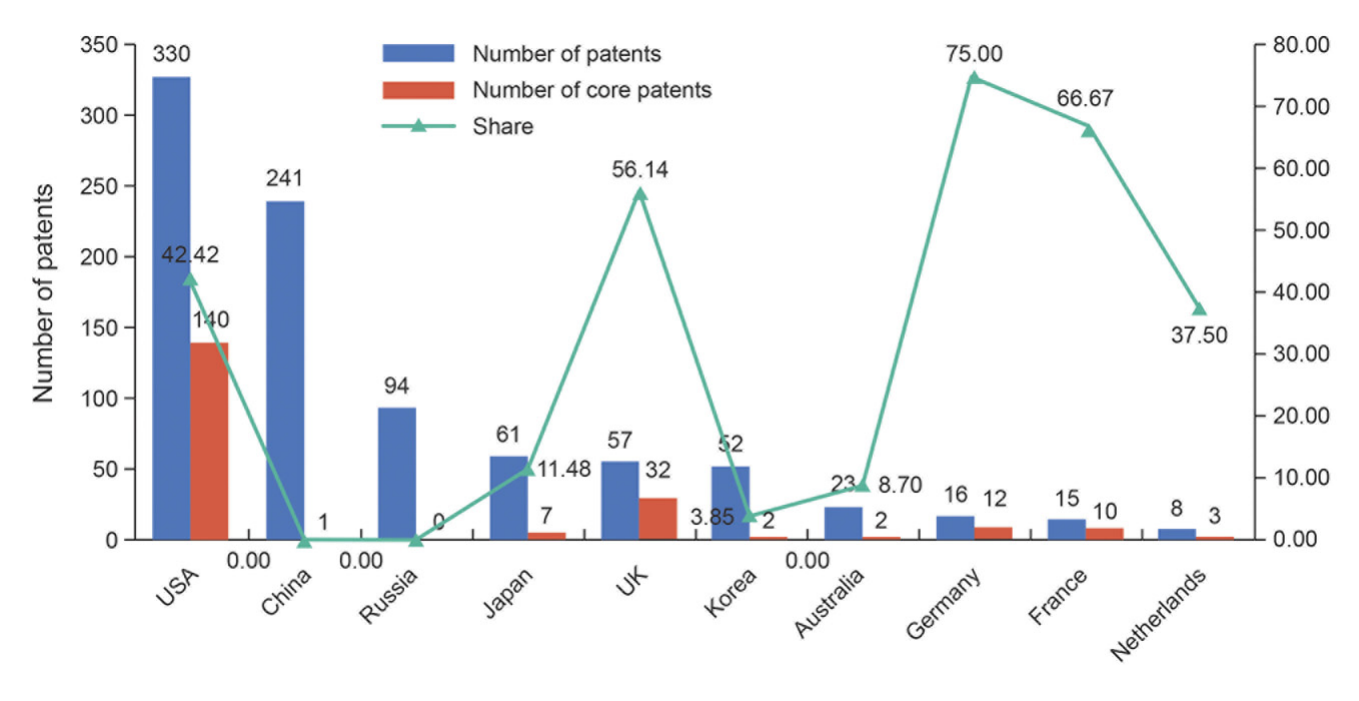
Numbers of patents and high-value patents in representative countries.

In addition, as showed in Fig 6, the current study makes a contrastive analysis of the distribution characteristics of the three types of patents in representative countries. We selected these countries based on the sort of national IVV patents ownership. It is worth mentioning that we regarded the top two countries/area code in the first priority number of patent as the nationality of the patent. As can be seen from the Fig 6 that the level of technological innovation in the global number of IVV patents Top10 countries is uneven: the US not only has the highest number of IVV patents, but also has the highest number of core patents and high-value patents, and the corresponding proportion is much higher than the world average level. In contrast, although China ranked second in the number of IVV patents, it only has one high-value patent and no core patent, which explained that its attention and inclination of science and technology policy and investment in recent years has effectively promoted the number of IVV patents, but there is still a great disparity between China and the leading level of the world in the quality of patents. Therefore, China needs more R&D investment to improve the overall level of IVV technology, and then makes up the gap with technology powerhouse. Russia, which is similar to China, lacks the influential patents; thus it cannot play a superb radiation and leading role. Besides, the four European countries (Britain, France, Germany, and Holland) account for 5–10 places in the ranking of number of IVV patents, and the proportion of their core patents and high-value patents in all is much higher than the world average, even higher than that of the US. Based on the above analysis, for the most part the applicants from European country applied for a patent through the EPO, we deem that the higher proportion may be associated with the more stringent quality requirements for patent application of EPO. In addition, South Korea and Australia are not ranked in the forefront of top 10 countries in terms of the quantity and quality of patents; therefore they need to strengthen the innovation investment in IVV further.

#### Comparison of four subfields of IVV

In this section, the general characteristics of the four subfields of IVV were compared, as showed in Table 8. From the relevant data on the number of IPC subgroups, it can be found that the average value of LAV is relatively small, and the value of SPV is larger than the other categories. Since LAV is the second generation of vaccine and has been investigated earlier, it is easier to attribute its technology innovations to a specific class of technology. Conversely, SPV is the product after the deep research on vaccine, and also is an outcome of discipline crossing between molecular biology and immunology, thus the number of IPC subgroups of SPV is larger than other categories. In addition, RV can be divided into three subcategories: recombinant protein vaccine, recombinant vector vaccine, and recombinant DNA vaccine. Despite the fact that recombinant DNA vaccine is the outcome of discipline crossing between immunology and genetics when they have developed to a certain extent, the correlative technology innovation of recombinant protein vaccine and recombinant vector vaccine is more remote and fundamental. On the whole, the feature of interdisciplinary is not obvious.

**Table 8:** Summary data statistics for IVV subfields patents.

	IV			LAV			RV			SPV		
	Max	Min	Mean	Max	Min	Mean	Max	Min	Mean	Max	Min	Mean
NIPC	39	1	6.52	17	1	3.81	59	1	5	39	1	6.32
NA	17	1	1.90	13	1	1.83	17	1	2.08	17	1	2.42
NCA	4	1	1.08	2	1	1.04	3	1	1.08	2	1	1.10
NI	23	1	4.13	10	1	3.58	17	1	3.83	17	1	4.21
NCI	3	1	1.12	6	1	1.09	3	1	1.12	2	1	1.05
CPP	64	0	2.05	39	0	1.34	82	0	1.41	35	0	6.57
FS	52	0	5.24	82	0	4.41	79	0	4.63	50	0	8.00

Data compiled by authors for this study.

NA = Number of applicants; NCA = Number of countries of applicants; NCI = Number of countries of inventors; NI = Number of inventors; NIPC = Number of IPC subgroup

The same features can also be reflected in the statistical indicators, including number of applicants, number of countries of applicants, number of inventors, and number of countries of inventors, which are associated with international cooperation. The value of LAV on these indicators is small while larger for SPV, IV and RV in between. One of the reasons is that more interdisciplinary follows more applicants and inventors participate in technology innovation, so the feature of indexes about interdisciplinary and cooperation is approximate. The other reason is that with the speeding of internationalization, technology innovation shows more and more characteristics of international at the present stage. Thus, the feature of internationalization were not evident in earlier research categories such as IV, whereas for the newer fields, like SPV and recombinant DNA vaccine, international cooperation is very frequent at the time of carrying on technology innovation, so the value is larger. In general, there is a certain correlation between statistical indicators of international cooperation and study period.

Through the observation of CPP and FS, which can reflect the patent value and influence, we can see a positive correlation between these two indicators, which means bigger CPP indicates bigger FS in this category. So the four types can be given an entirely consistent order based on the sorting of CPP and FS value in numerical, i.e., SPV > IV > RV > LAV. This also to some extent explains the current heat and attention sort about study. As a main direction of the new vaccine to prevent and control the infectious diseases and malignant tumor, SPV is also an ideal new the safety vaccine. With the advances in technology, technology innovation about this category may have a great progress in the future.

**Fig 7:**
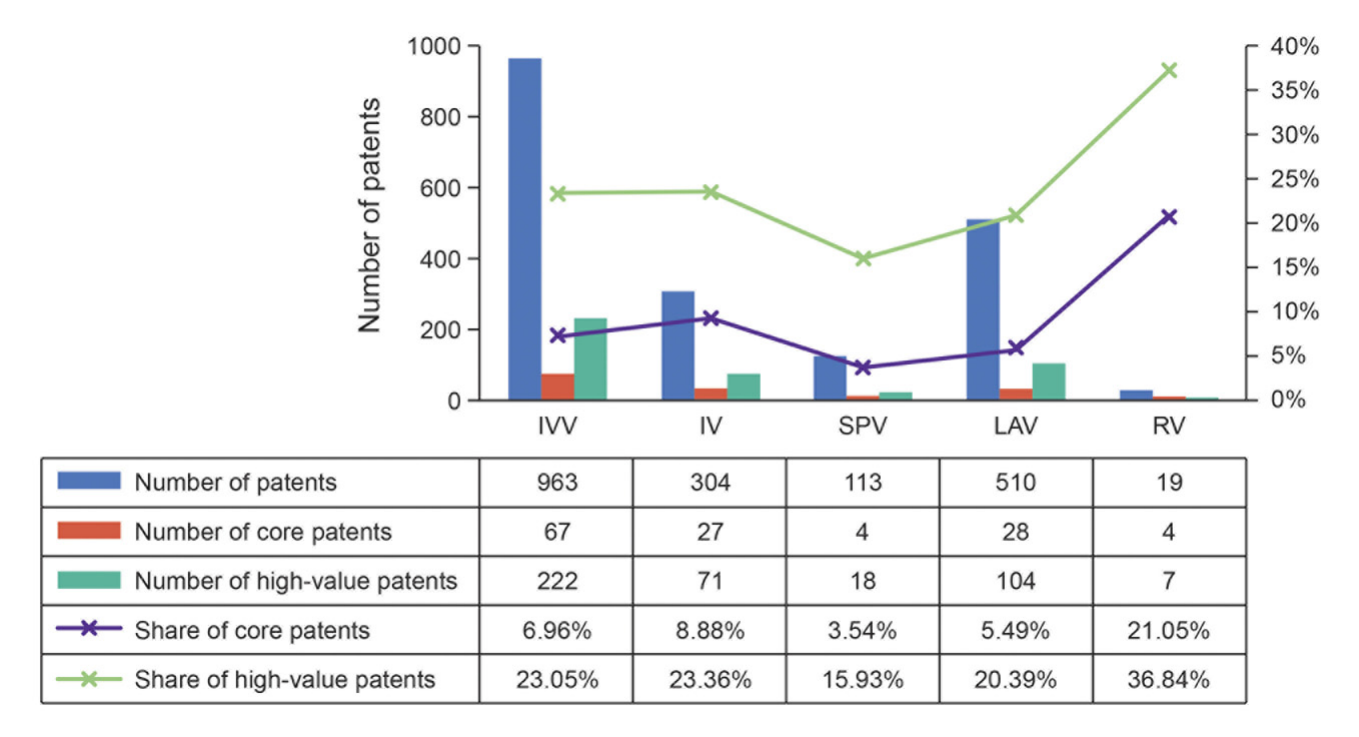
Numbers of patents, core patents, and high-value patents of IVV subfields.

It is evident from Fig 7 that the rankings of share of core patents and high-value patents for four categories are consistent with the sorting based on CPP and FS in the above paragraph, and further substantiate the observation of a positive correlation between CPP and FS. SPV category is much higher than the other categories of the patent value and influence, which further sustains the prediction that SPV will develop rapidly in the future.

## Discussion

The technological information in patent documents provides a useful tool to the studies of a particularly technology area. This study is a most comprehensive scientometric analysis of the IVV field. Following conclusions are drawn from the longitudinal analysis of IVV patents.

Although studies of the IVV proliferate in recent years, the resource distribution status of global IVV is still uneven. The governments, enterprises, universities, and research institutions have devoted to the scientific advancement, technology development, and product innovation of IVV; however, the overall growth trend of IVV patent data shows a fluctuating pattern. What is more, the IPC distribution of IVV technical topics is relatively concentrated, and each category has respective focus on different IPC subgroup. At the national level, the US is not only worthy of the world leader in terms of new patent applications, but also a more intense competition area in the market of IVV technology. European countries have a number of the world's leading vaccine companies, whose patent layout is global. In contrast, Chinese patent’s application and acceptance quantities are both at the forefront level. However, the patents granted by SIPO are mainly from Chinese applicants, and the amount of Chinese IVV patents granted by other countries or organizations is relatively small. In the context of existing evaluation system for scientific research, Chinese patents always are characterized as quantity-adding focusing but quality-improvement ignoring. However, various countries have launched an international competition in this field and the competition may be more intense in the future. Therefore, China should improve the overall level of IVV technology, and make up the gap between China and the other technological powers.

The multi-developing feature of variety has emerged in global IVV field, but it inevitably brings about severe challenges. Firstly, the value and impact of a patent are connected with the number of IPC subgroup of the patent; in other words, to some extent, the merging of IVV technology can affect the value and impact of patents, which verifies a greater diversity of technology topics, as mentioned above. Secondly, there is no significant difference between core patents, high-value patents, and all patents on the number of applicants and inventors; but effective international communications and cooperation can promote the generation of high-value and high-impact patents in terms of countries of patent applicants and inventors. Last but not least, it can be seen that there is some positive correlation between core patents and high-value patents; and core patents have higher valuation while high-value patents have higher influence.

Finally, several points should be noted in the analysis of the different development characteristics of four categories of IVV. On one hand, since earlier LAV studied is more basic than SPV in technology innovation, it is easier to boil down to a kind of technology. Conversely, SPV is the product after the intensive research in vaccine and is also the outcome of a discipline crossing between molecular biology and immunology when they have developed to some extent. Thus, it is similar to the recombinant DNA vaccine, and the correlative technology innovation of recombinant protein vaccine and recombinant vector vaccine is more remote and fundamental, thus the feature of interdisciplinary RV is not obvious, on the whole. On the other hand, the early or late beginning time of research is concerned with the frequency of international cooperation. And the four subfields can be given a completely consistent order based on the sorting of CPP and FS value in numerical, i.e. SPV, IV, RV, LAV, which are also consistent with the rankings of share of core patents and high-value patents for the four categories. Accordingly, we speculate that the synthetic peptide vaccine may have a greater development in the future with technology advancements.
